# Smartphone-based holographic measurement of polydisperse suspended particulate matter with various mass concentration ratios

**DOI:** 10.1038/s41598-022-27215-6

**Published:** 2022-12-30

**Authors:** Jihwan Kim, Youngdo Kim, Kyler J. Howard, Sang Joon Lee

**Affiliations:** 1 Department of Mechanical Engineering, Pohang University of Science and Technology, Pohang, 37673 Republic of Korea; 2 School of Biomedical Engineering, Colorado State University, Fort Collins, CO 80521 USA

**Keywords:** Applied optics, Imaging and sensing, Microscopy, Optofluidics

## Abstract

Real-time monitoring of suspended particulate matter (PM) has become essential in daily life due to the adverse effects of long-term exposure to PMs on human health and ecosystems. However, conventional techniques for measuring micro-scale particulates commonly require expensive instruments. In this study, a smartphone-based device is developed for real-time monitoring of suspended PMs by integrating a smartphone-based digital holographic microscopy (S-DHM) and deep learning algorithms. The proposed S-DHM-based PM monitoring device is composed of affordable commercial optical components and a smartphone. Overall procedures including digital image processing, deep learning training, and correction process are optimized to minimize the prediction error and computational cost. The proposed device can rapidly measure the mass concentrations of coarse and fine PMs from holographic speckle patterns of suspended polydisperse PMs in water with measurement errors of 22.8 ± 18.1% and 13.5 ± 9.8%, respectively. With further advances in data acquisition and deep learning training, this study would contribute to the development of hand-held devices for monitoring polydisperse non-spherical pollutants suspended in various media.

## Introduction

With the increasing concerns about global environmental pollution, real-time monitoring of particulate matter (PM) suspended in the air or water has emerged as an important requisite for environmental quality control^1–3^. Suspended PMs are fine, water-insoluble particles, such as lithogenic materials, heavy metals, and pollutants floating in the air or water. Airborne PMs consisting of heterogeneous chemical compounds^4^ can easily penetrate into the human body, causing severe health problems^5–7^. In water, suspended PMs can bring about adverse effects on aquatic ecosystems^8^, such as developmental toxicity^9^ and light attenuation that hinders photosynthesis and nutrient uptake of aquatic organisms^10^. Therefore, precise monitoring of suspended PMs is needed to prevent its adverse effects on public health and nature.

Measurement of particle size is one of the essential analyses that are widely utilized in industrial, biological, and biomedical fields^11–15^. Several techniques have been developed to measure the concentrations and spatial distributions of physical sizes of solid particles suspended in a medium. Gravimetric measurement adopts filters with varying pore diameters to classify solid particles^16^. Sedimentation field flow fractionation can classify particle sizes by using their differences in settling velocities in a flow^17^. Laser diffraction method has also been commonly adopted for analyzing particle size based on the Fraunhofer diffraction theory^18^. The scattering intensity of light is proportional, while its scattering angle is inversely proportional, to particle size. Ultraviolet–visible spectroscopy measures the light absorption rate depending on the wavelength (*λ*) of an incident light and provides information of the PM sample, including molecular composition and concentration^19^. Nanoparticle tracking analysis^20,21^ and dynamic light scattering^22,23^ calculate the diameters of submicron particles based on their Brownian motions and the Stoke-Einstein equation. For dynamic light scattering, the intensity-weighted particle size distribution (PSD) can be converted into volume- and number-weighted PSDs if the optical characteristics of the test particles, media, and reference materials are known in advance^24–26^. However, these techniques usually require expensive and specialized instruments to obtain accurate information.

Digital holographic microscopy (DHM) is an effective 3D imaging technique for volumetric measurement of various micro-scale particles, such as microorganisms^27^, microplastics^28^, and PMs^29,30^. Conventional DHM techniques use numerical reconstruction and autofocusing algorithms to extract 3D locational and morphological information of test particles from recorded 2D holographic images^31–33^. However, these techniques require advanced optical alignment, high computational cost, and large data storage. With the aid of the rapidly growing artificial intelligence (AI) technology^34^, DHM has been extensively combined with AI to overcome the technical limitations of conventional techniques to improve spatial resolution, reduce noises, and minimize computational cost^35–38^. For a cost-effective device for quantitative analysis of various particulates, smartphone-based DHM (S-DHM) combined with AI technology has been investigated for biomedical diagnosis and environmental monitoring^39–41^. In our previous study, a compact S-DHM-based device for high PM concentration measurement was developed based on holographic speckle patterns of PMs and deep learning^41^. Holographic speckle patterns vary depending on the optical characteristics and concentrations of test particles^42,43^. The mass concentrations of airborne PMs can be directly predicted by training deep learning algorithms with their holographic speckle images and the corresponding PM concentration labels. However, this technique requires further improvement in digital image processing and deep learning network to obtain accurate measurements of polydisperse PMs.

In this study, a real-time, non-invasive, and cost-effective device is proposed to measure the concentrations of highly concentrated polydisperse PMs suspended in water. This hand-held device is developed by integrating a S-DHM system and a deep learning network called Holo-SpeckleNet^41^. The developed S-DHM is used to acquire holographic speckle patterns of PMs, consisting of various mass concentration ratios of coarse and fine PMs (PM_c_ and PM_f_, respectively). The recorded holographic images are converted into trainable datasets by applying optimized digital image processing. The deep learning network which consists of a deep autoencoder (DAE) and regression layers is trained using the converted speckle images of monodisperse PM_c_ and PM_f_ samples and the corresponding ground truth concentration labels. The network trained with the monodisperse PM samples can selectively predict PM_c_ and PM_f_ concentrations from the speckle images of polydisperse PM mixture without time-consuming computational procedures required in conventional DHM techniques. The proposed S-DHM-based device can be effectively utilized to measure the absolute mass concentrations of polydisperse PMs with cost-effective optical components (~ $390) and low computational cost for training procedure.

## Methods

### Sample preparation

Arizona test dust (ISO 12103-1) is used to make the PM test samples. The average diameters of PM_f_ and PM_c_ are 1.256 ± 1.309 µm and 7.657 ± 1.286 µm, respectively. The size distributions of PMs are measured by using the Multisizer 3 particle counter (Beckman Coulter, USA). The polydispersity index (PDI) is defined as the square of mean diameter of PMs divided by their standard deviation. The PDI values of PM_f_ and PM_c_ are 1.086 and 0.0282, respectively. Two reference samples of PM_f_ and PM_c_ with mass concentration values of 20 µg/ml are prepared by mixing PMs and distilled water. 20 mg of PM is measured by using an electronic balance (CAS, Korea) and then mixed with 1L of distilled water in a 1L wide neck bottle (Azlon, UK) to make two reference samples of PM_f_ and PM_c_. The two reference samples and distilled water are then mixed in 30 ml square-shaped transparent bottles (Triforest, USA) at different volume ratios using 10 ml sterile syringes (Shinchang Medical, Korea) to prepare PM test samples with various concentration ratios of PM_f_ and PM_c_.

### S-DHM-based device for measuring polydisperse PM

**Figure 1:**
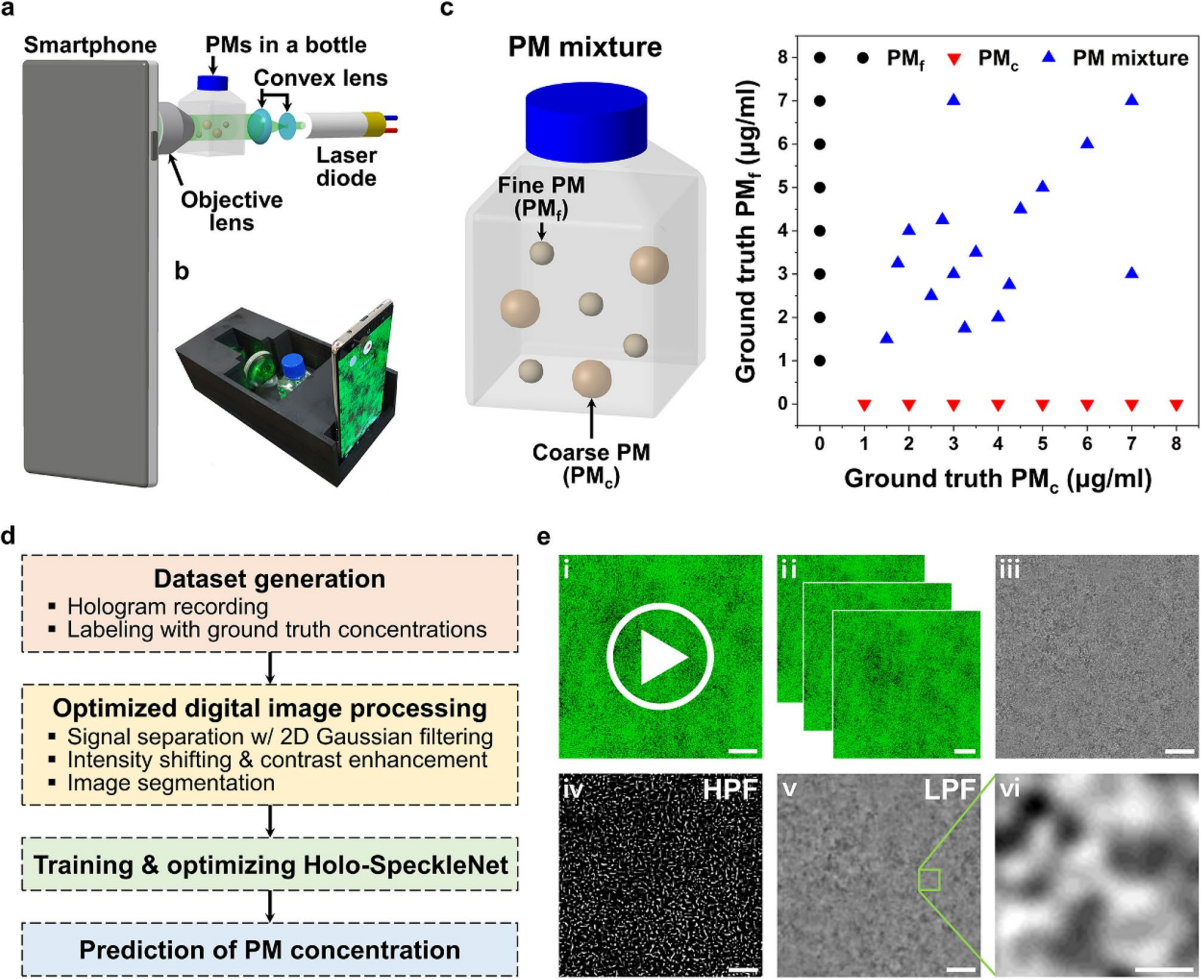
Smartphone-based device for monitoring suspended PMs in water. (**a**) Schematic of the experiment setup with optical arrangement. (**b**) Photograph of the S-DHM device. (**c**) Suspended PMs in a bottle. (**c**) PM test samples with various mass concentration ratios of PM_f_ and PM_c_. (**d**) Overall procedure for predicting PM concentrations from holographic speckle images. (**e**) Digital image processing techniques used for preprocessing holographic speckle images. Scale bars: (**e**_**i–v**_) 25 μm; (**e**_**vi**_) 5 μm.

Figure 1a shows a schematic of the proposed S-DHM system used for measuring suspended PM particles. A coherent beam is generated from a laser diode (*λ* = 532 nm, 20 mW) connected to a rechargeable lithium-ion battery (4.5 V). The laser beam is expanded to make a quasi-uniform collimated beam with the aid of an aspheric lens (*f* = 3.1 mm) and a plano-convex lens (*f* = 40 mm). Holographic speckle pattern is magnified by using a 20 × objective lens (NA = 0.4, *f* = 9 mm, Newport, USA). Holograms of PM particles are then recorded by using a Samsung Galaxy S10 + smartphone (Samsung, Korea). Consecutive holograms are recorded within 0.4 s by adopting the super-slow-motion mode (1280 × 720 pixels; 960 fps) of the smartphone of which the magnified pixel size is 250 nm. The built-in back camera module of the smartphone is slightly modified by removing the auto-focusing lens to avoid spherical aberration. The aligned optical components are mounted on a 3D printed housing (242 mm × 110 mm × 70 mm) (Fig. 1b).

The test samples are the mixtures of PM_c_ and PM_f_ (Fig. 1c). PM_c_ has particle diameters in the range from 5 to 10 μm, while PM_f_ has particle diameters less than 3 μm. PM_c_ and PM_f_ are suspended in water contained in 30 ml bottles with mass concentrations in the range of 1–8 µg/ml to produce monodisperse PM_c_ and PM_f_ samples, respectively. These two types of PM particles are mixed in the bottles to prepare polydisperse PM samples with various concentration ratios. Because each test sample is placed close to the objective lens, the focal plane of the lens is located 5 mm away from the wall of the bottle within the volume of water. The prepared test samples are used to acquire holographic speckles of monodisperse and polydisperse PM particles.

Figure 1d shows the overall procedure of the proposed S-DHM-based technique for measuring suspended PM particles. Holograms and the corresponding ground truth concentration labels of PM particles are acquired at first. The optimized digital image processing technique is then applied to convert the recorded holograms into trainable datasets. The converted images and the corresponding ground truth PM concentrations are classified in pairs into the training, validation, and test datasets. These datasets are used to train and optimize the artificial neural network composed of a DAE^44^ and three regression layers. Finally, the PM_c_ and PM_f_ concentrations are evaluated from the acquired holograms of PM samples.

Raw holographic speckle images are converted into trainable datasets after applying the digital image processing (Fig. 1e). Holographic speckle patterns of test samples are recorded with the super-slow-motion mode of the smartphone (Fig. 1e_i_; .mp4; 1280 × 720; 960 fps). Each recorded video file is separated into sequential RGB holograms (Fig. 1e_ii_; .tiff). Each RGB hologram is cropped and converted into grayscale (Fig. 1e_iii_; .tiff; 700 × 700). In each hologram, background noises are commonly formed due to unintended external vibrations and dust particles attached on optical components. A background image is obtained by ensemble averaging of consecutive holographic images. Then, the signal-to-noise ratio (SNR) of the holographic speckle patterns of test samples is enhanced by subtracting spatially invariant background noises.

The holographic speckle patterns of polydisperse PM samples are composed of speckle signals of PM_c_ and PM_f_. Each speckle signal in a recorded hologram can be separated by the difference in their spatial frequencies. First, a 2D fast Fourier transform (2D FFT) is used to convert a hologram from the spatial domain to frequency domain. 2D Gaussian pass filter is then adopted to eliminate the high or low spatial frequency components in the frequency domain. The Gaussian low-pass filter (LPF) and high-pass filter (HPF) are defined as follows:

1 $${\text{LPF}}(u,v) = \,\,e^{{\frac{{ - \,\,(\,u^{2} + v^{2} )}}{{4R^{2} }}}}$$

2 $${\text{HPF}}(u,v) = \,\,1 - {\text{LPF}}(u,v)$$

where *R* is the filter size, and *u* and *v* are the coordinates in the frequency domain. The spatial frequency of PM_f_ speckles is usually higher than that of PM_c_ speckles. Therefore, PM_f_ and PM_c_ speckles can be selectively extracted from the original hologram by applying the HPF and LPF, respectively. Thereafter, an inverse 2D FFT is used to convert the filtered image from the frequency domain to the spatial domain (Fig. 1e_iv,v_). Thereafter, the intensity shifting and contrast enhancement methods are applied to emphasize the representative features of holographic speckle patterns. Each hologram is then segmented into 10 × 10 pieces with a physical dimension of 70 × 70 pixels to prevent data dissipation of small speckle signals by enlarging their relative sizes compared with segmented image sizes (Fig. 1e_vi_). Without image segmentation, the information of small speckle signals recorded in raw holograms is easily dissipated during data compression in the deep learning training process. Therefore, the relative sizes of speckle signals should be sufficiently enlarged to get high prediction accuracy (Fig. S1). The processed images and the corresponding ground truth PM concentration information are classified into the training, validation, and test datasets. Each class of training, validation, and test datasets consisting of 10,000, 20,000, and 30,000 segmented images is generated from 100, 200, and 300 sequential holograms, respectively.

### Calculation of optical characteristics of holographic speckle patterns

Optical characteristics of six different datasets, including the high-pass filtered and low-pass filtered speckle images of monodisperse PM_f_, monodisperse PM_c_, and PM mixture are investigated. First, the intensity gradient is given by the variations in the pixel intensity (*I*) between adjacent pixels^45^. The mean intensity gradient of the spackle pattern recorded in an image is calculated as follows:

3 $$\nabla I_{mean} = \sum\limits_{x = 1}^{M - 1} {\sum\limits_{y = 1}^{N - 1} {\sqrt {\left( {I(x + 1,y) - I(x,y)} \right)^{2} + \left( {I(x,y + 1) - I(x,y)} \right)^{2} } } } /((M - 1) \times (N - 1))$$

where *x* and *y* represent the discrete image coordinates with *M* × *N* pixels. The pixel intensity is distributed between zero (black) to one (white) in an image. Second, the speckle size is the average pixel area occupied by the speckle pattern in the image^46^. The normalized intensity distributions of low-pass filtered and high-pass filtered holograms of monodisperse PM_f_ and PM_c_ are shown in Fig. S2. The average and standard deviation of pixel intensities contained in each case are calculated. The intensity threshold level is defined as the sum of the average and standard deviation of pixel intensities. The holographic images are then binarized using the intensity thresholding method. The “nnz” and “bwconncomp” functions of the MATLAB software are then utilized to extract the total pixel area and the total number of speckle pattern in each binarized image, respectively. Thereafter, the mean speckle size is obtained by dividing the total pixel area with the total number of speckles.

Third, the speckle width represents the mean thickness of speckle patterns calculated with the normalized autocovariance function from the intensity distribution in the image^47^. The normalized autocovariance function is defined as follows:

4 $$f(x,y) = \frac{{FFT^{ - 1} \left[ {\left| {FFT\left[ {I(x,y)} \right]^{2} } \right|} \right] - \left\{ {\sum\nolimits_{x = 1}^{M} {\sum\nolimits_{y = 1}^{N} {I(x,y)/(M \times N)} } } \right\}^{2} }}{{\left\{ {\sum\nolimits_{x = 1}^{M} {\sum\nolimits_{y = 1}^{N} {I(x,y)^{2} /(M \times N)} } } \right\}^{2} - \left\{ {\sum\nolimits_{x = 1}^{M} {\sum\nolimits_{y = 1}^{N} {I(x,y)/(M \times N)} } } \right\}^{2} }}$$

where *FFT* and *FFT*^−1^ are the fast Fourier transform (FFT) and inverse FFT, respectively. The speckle width is evaluated from the full width at half maximum (FWHM) of the normalized *f*(*x*,*y*). Thereafter, the horizontal and vertical widths are averaged to obtain the mean speckle width contained in the image. Forth, the 2D spatial frequency distributions of speckle patterns are acquired by applying the FFT function on the speckle images^33^. The power spectral density profiles of horizontal and vertical spatial frequency components are obtained from the frequency domain of the transformed array. The mean spatial frequency is then calculated by averaging the mean horizontal and vertical spatial frequencies.

### Hyperparameters of deep learning algorithms

Due to the limited performance of an embedded smartphone central processing unit (CPU), the number of neurons consisting of DAE is optimized to maximize PM measurement accuracy and minimize computational cost. More detailed spatial features of speckle pattern are extracted from input images with the increase of the number of neurons, while the computational cost also largely increases. The optimum size of DAE is investigated to effectively extract the common traits of speckle patterns with varying sizes and concentrations of PMs. Therefore, the DAE structure is composed of an encoder (4900 × 512 × 256 × 128 × 64 neurons) and a decoder (64 × 128 × 256 × 512 × 4900 neurons). The Adam optimizer and sigmoid transfer function are used to minimize the root mean square error (RMSE) between the input and reconstructed images. The RMSE is calculated by averaging pixelwise residuals between the two images, and obtaining the square root of the mean. The batch size, epochs, and learning rate of DAE are set to 4096, 5000, and 10^−3^, respectively. The regression layers (64 × 256 × 256 × 1 neurons) are trained with the main features extracted from the latent space of DAE and the corresponding PM concentration labels. Gradient descent optimizer and rectified linear unit activation function are utilized to minimize the mean absolute error (MAE) between the PM prediction values and PM concentration labels. The MAE is calculated by averaging absolute errors between the predicted and ground truth values. The batch size, epochs, and learning rate of the regression layers are set to 8192, 10,000, and 10^−7^, respectively. The weights and biases in the deep learning algorithms are iteratively updated to find the global minima of loss functions based on a stochastic gradient descent method.

### Development environment

Deep learning algorithms are trained on a desktop computer composed of Nvidia GeForce RTX 3090 GPU, AMD Ryzen 5950 X CPU, and 128 GB RAM. The integrated development environment consists of Python 3.6.7, Anaconda3-4.5.11, PyCharm (JetBrains, Czech Republic), TensorFlow-gpu 2.4.1, NVIDIA CUDA toolkit 10.0, and cuDNN 7.4.2. The contrast of speckle pattern images is enhanced using the Python imaging library. MATLAB R2021a software is utilized to quantitatively analyze various optical characteristics of speckle patterns. Statistical data analyzed by using ANOVA are expressed as the mean ± standard deviation.

## Results

### Optical characteristics of holographic speckle patterns

**Figure 2:**
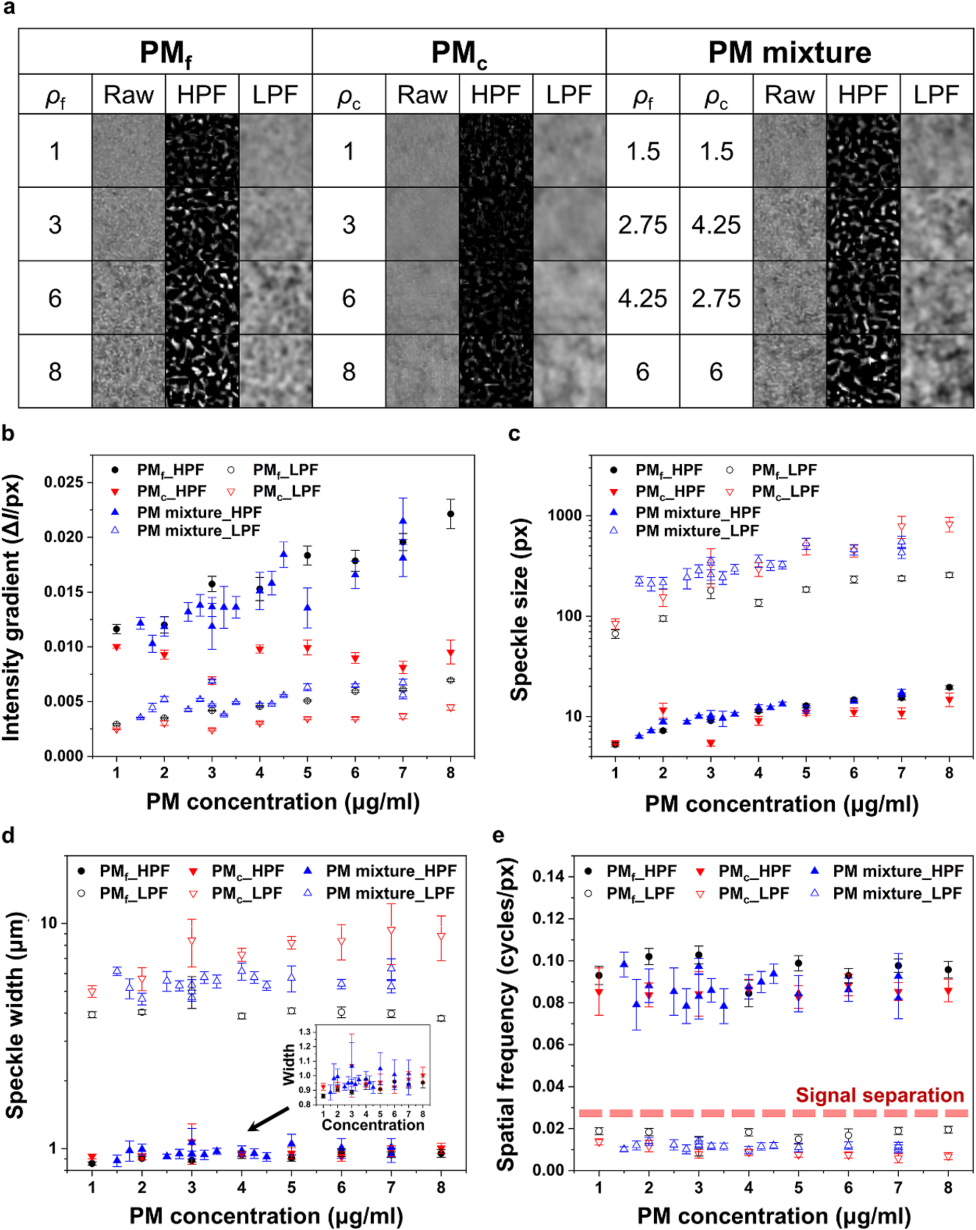
Analyses of various optical characteristics of holographic speckle patterns. (**a**) Typical images of raw, high-pass filtered, and low-pass filtered holographic speckle patterns according to their PM concentration. Comparisons of optical characteristics of holographic speckle patterns: (**b**) mean intensity gradient, (**c**) speckle size, (d) speckle width, and (e) spatial frequency. The dotted red line (*y* = 0.0286) denotes the signal separation criterion with the Gaussian filter size *R* of 20. Segment sizes of speckle images: (Raw, LPF) 35 × 35 μm^2^; (HPF) 17.5 × 17.5 μm^2^.

A collimated incident laser beam is scattered by a test particle and the scattered beam interferes with a reference beam. Interference fringe pattern varies depending on the physical properties of the test particle, such as its size, shape, refractive index, and distance from the focal plane. In a turbid medium with highly-concentrated suspended particles, numerous interference fringe patterns are generated. Multiple scattering phenomena occur owing to the presence of adjacent particles and holographic speckle patterns are formed^43^. Holographic speckle pattern largely depends on the physical properties of test particles suspended in a volume. Figure 2a shows typical holographic speckle patterns according to the ground truth concentration values of PM_f_ (*ρ*_f_) and PM_c_ (*ρ*_c_). Various optical characteristics of PM speckles according to their concentration are quantitatively analyzed, including mean intensity gradient^45^, speckle size^46^, speckle width^47^, and spatial frequency^33^ (Fig. 2b–e, Tables S1–4). The *x*-axes of monodisperse PM_f_ (PM_f__HPF, PM_f__LPF) and high-pass filtered PM mixture (PM mixture_HPF) speckles stand for PM_f_ concentration, while those of monodisperse PM_c_ (PM_c__HPF, PM_c__LPF) and low-pass filtered PM mixture (PM mixture_LPF) speckles represent PM_c_ concentration. The filter size *R* is set as 20. The intensity gradient and size of speckles tend to increase with PM concentration. On the other hand, the intensity gradient of high-pass filtered speckles of monodisperse PM_c_ shows no relationship with PM concentrations. Speckle width of high-pass filtered datasets and the corresponding PM concentration exhibit a positive correlation with large deviations. Among the low-pass filtered speckles, only monodisperse PM_c_ dataset shows a positive relationship between its speckle width and PM concentration. The speckles filtered with HPF and LPF largely differ in their spatial frequencies.

### Prediction of PM_f_ concentrations

**Figure 3:**
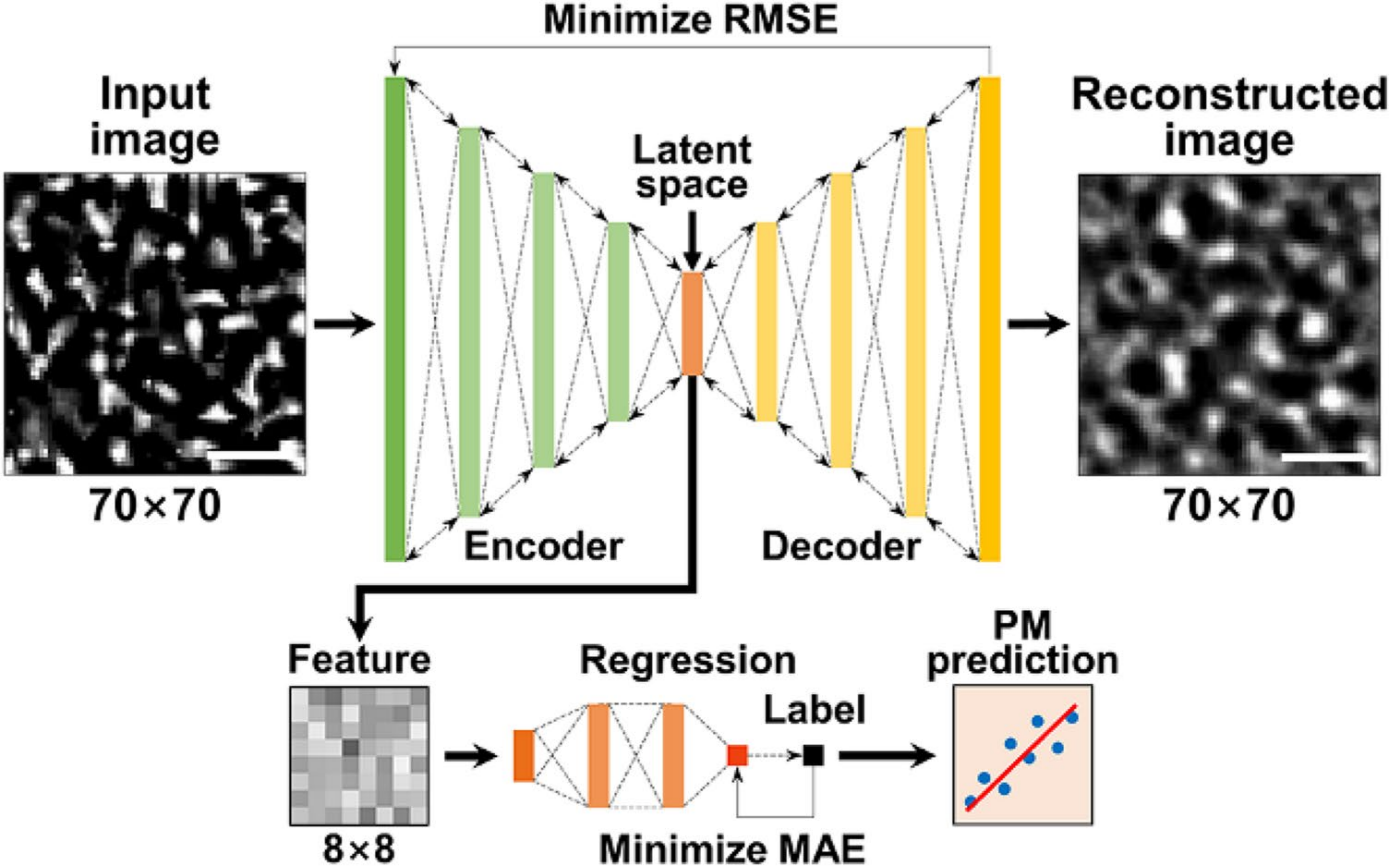
Architecture of the deep learning algorithms used for feature extraction and regression analysis. Scale bars are 5 μm.

The variations in optical characteristics of PM speckles in accordance with the size and concentration of PM samples is trained by Holo-SpeckleNet^41^ illustrated in Fig. 3. A processed speckle image (70 × 70 pixels) is flattened and fed to an encoder and compressed to extract main features in the latent space. The extracted features are then reconstructed into the original size by a decoder. After minimizing the RMSE between the input and reconstructed images, 64 main features of the input image are obtained. The obtained main features and the corresponding PM concentration labels are fed to the regression layers and trained by minimizing their MAE. The trained model is found to rapidly predict the PM concentration of the input image. The detailed network structure and mathematical equations of Holo-SpeckleNet are explained in our previous study^41^.

**Figure 4:**
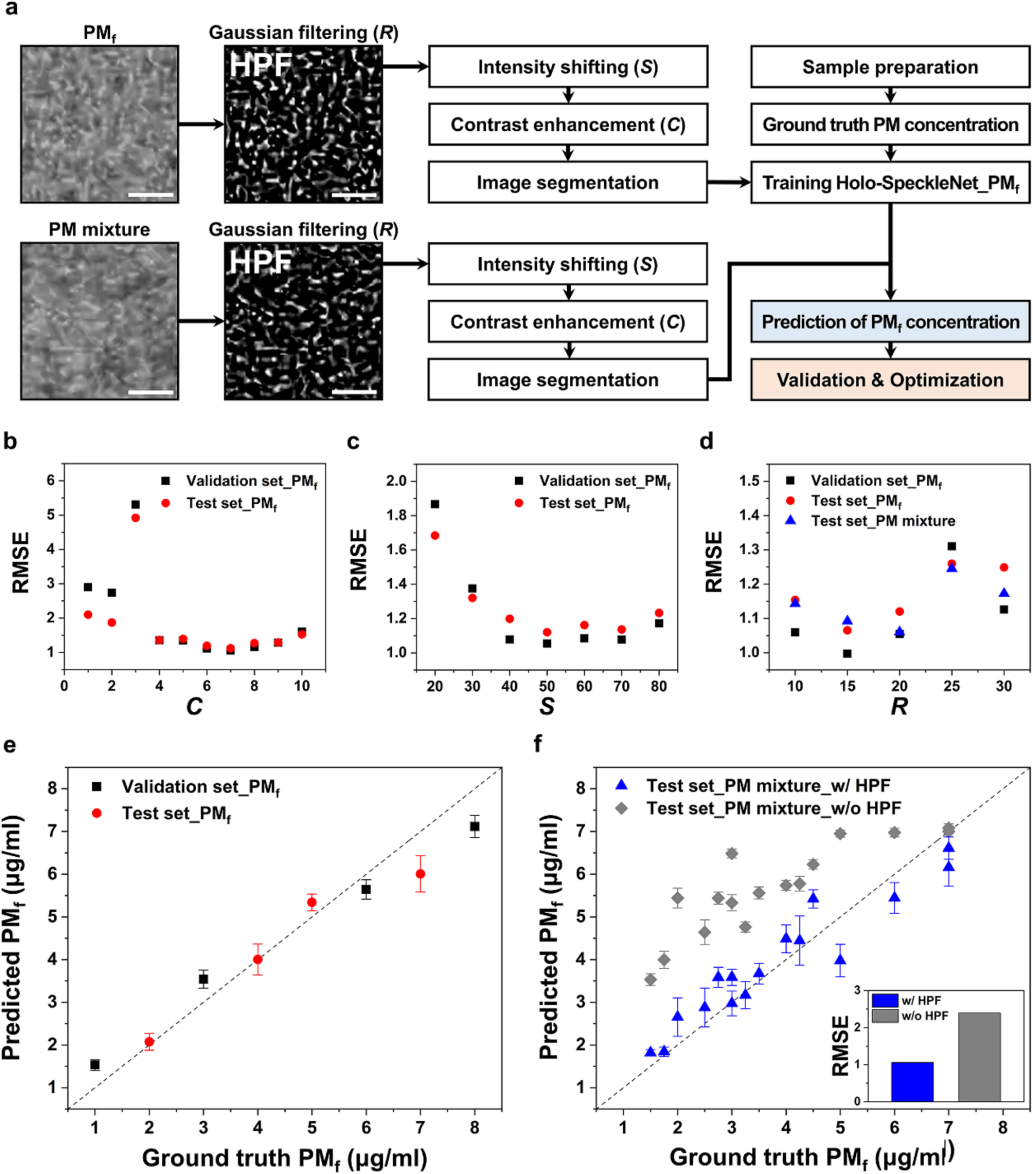
Smartphone-based measurement of PM_f_ concentrations. (**a**) Overall workflow used to predict PM_f_ concentrations. PM concentration labels of segment images: (PM_f_) *ρ*_f_ = 8 µg/ml; (PM mixture) *ρ*_f_, *ρ*_c_ = 6 µg/ml. Optimization of hyperparameters associated with digital image processing: RMSE variations according to (**b**) contrast enhancement parameter *C*, (**c**) intensity shifting parameter *S*, and (**d**) Gaussian filter size *R*. Predicted PM_f_ concentrations of (**e**) monodisperse PM_f_ and (**f**) polydisperse PM mixture samples. Scale bars are 10 μm.

Figure 4a shows the flow chart of PM_f_ concentration measurement. A gray-scale speckle image of monodisperse PM_f_ is filtered with HPF. The intensity distribution of the high-pass filtered image is biased to black color (pixel intensity = 0). Therefore, the intensity distribution is shifted to white color (pixel intensity = 255) by intensity shifting parameter *S* to enhance the deep learning training performance. Contrast enhancement and image segmentation are then applied to acquire trainable datasets. The deep learning network is trained with the processed PM_f_ speckles and the corresponding labels. PM_f_ concentration labels of 1, 3, 6, and 8 µg/ml are used as the training datasets. After training, the identical digital image processing is applied to speckle images of PM mixture to predict their PM concentrations.

Hyperparameters related with the Gaussian filtering (*R*), intensity shifting (*S*), and contrast enhancement (*C*) are optimized to minimize the RMSE of test datasets (Fig. 4b–d). The optimum value of one hyperparameter is found by iteratively changing it while other hyperparameters are fixed with their optimum values. The optimum *S* and *C* values are found to be 50 and 7, respectively. For the Gaussian filtering, the optimum *R* values of monodisperse PM_f_ and PM mixture are 15 and 20, respectively. The Gaussian filter size *R* is set to 20, because the main objective of this study is to measure the concentrations of polydisperse PMs. The mean PM concentration value for one input image is obtained by averaging the predicted PM concentration values of 100 segments of the input image. For each class, the mean PM concentrations of sequential images are averaged again to acquire the final predicted value of the class. As a result, the trained model can predict PM_f_ concentrations with a measurement error of 14.1 ± 16.7% (Fig. 4e, Table S5). The PM_f_ concentrations of PM mixture are also predicted with a measurement error of 13.5 ± 9.8% (Fig. 4f, Table S6). Since the PDI of PM_f_ samples is relatively higher than that of PM_c_, additional measurement errors might be induced by the variation in the size of PM_f_ suspended in the monodisperse PM_f_ and PM mixture samples. With the aid of HPF, the RMSE of predicted the PM_f_ concentrations decreases by approximately 55.7%. These results imply that the speckle signal of PM_f_ can be effectively extracted from that of PM mixture using HPF.

### Prediction of PM_c_ concentrations

**Figure 5:**
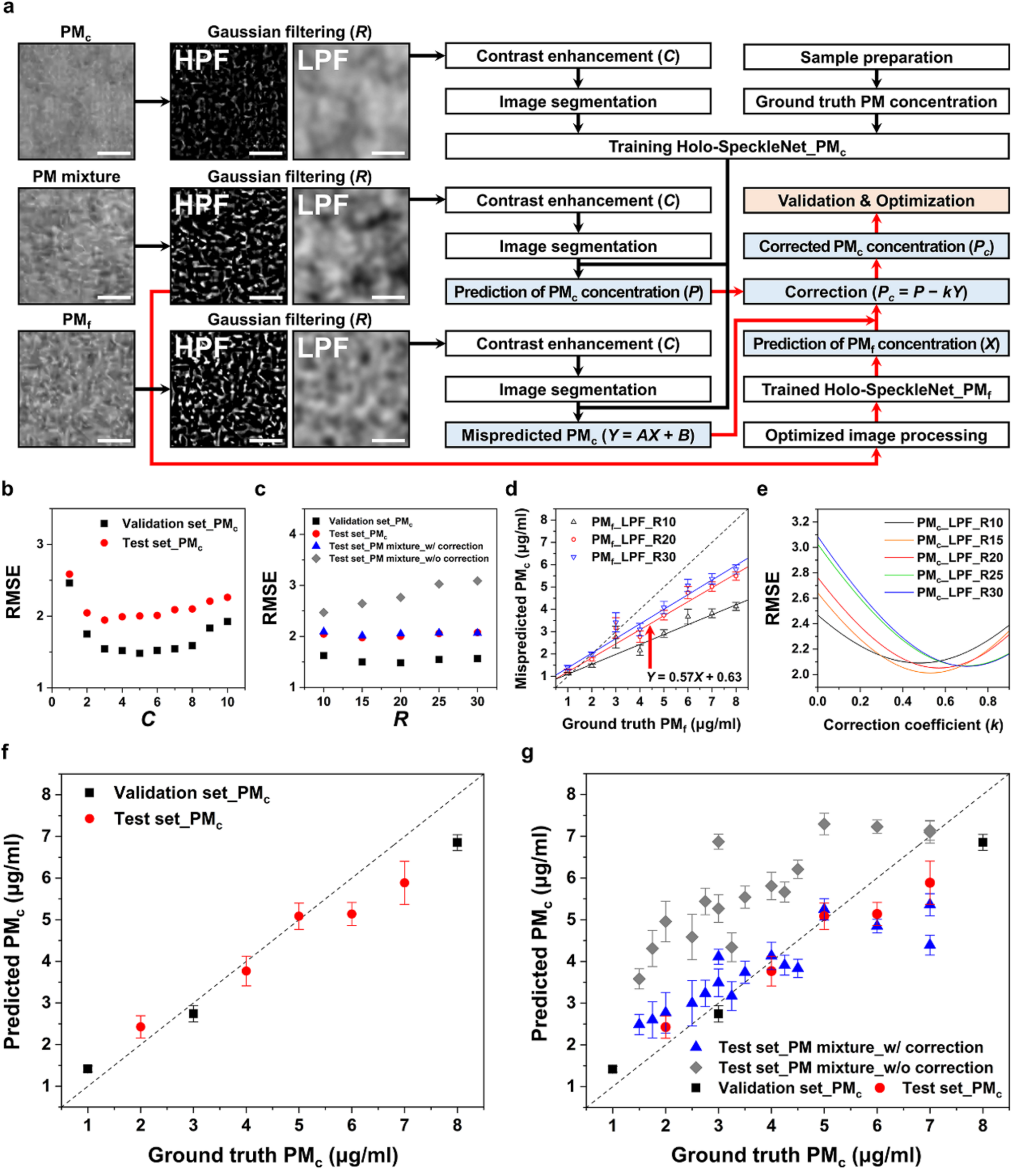
Smartphone-based measurement of PM_c_ concentrations. (**a**) Overall workflow used to predict PM_c_ concentrations. PM concentration labels of segment images: (PM_f_, PM_c_) *ρ*_f_, *ρ*_c_ = 8 µg/ml; (PM mixture) *ρ*_f_, *ρ*_c_ = 6 µg/ml. Optimization of hyperparameters associated with digital image processing: RMSE variations according to (**b**) contrast enhancement parameter *C* and (**c**) Gaussian filter size *R*. (**d**) Mispredicted PM_c_ concentrations of monodisperse PM_f_ holograms filtered by LPFs with the Gaussian filter size *R* of 10, 20, and 30. (**e**) Optimization of correction coefficient (*k*) used in the correction procedure of mispredicted PM_c_ concentrations. Predicted PM_c_ concentrations of (**f**) monodisperse PM_c_ and (**g**) polydisperse PM mixture samples. Scale bars are 10 μm.

Figure 5a shows the flow chart of PM_c_ concentration measurement. The shifting parameter *S* is set to zero, because the mean pixel intensity of a low-pass filtered image is already placed around the middle of black and white color (pixel intensity = 128). After applying contrast enhancement and image segmentation procedures, the deep learning network is trained with the processed PM_c_ speckles and their corresponding labels. PM_c_ concentration labels of 1, 3, and 8 µg/ml are used as the training datasets. For monodisperse PM_c_, the optimum *C* values of the validation and test datasets are 5 and 3, respectively (Fig. 5b). The total RMSE of two datasets is minimized at *C* = 3, while the prediction accuracy in the concentration range of 1–2 µg/ml is better at *C* = 5 (Fig. S3). Therefore, the optimum *C* is set to 5. The optimum *R* values of the validation and test datasets are 20 and 15, respectively (Fig. 5c). The optimum *R* is set to 20, which provides a high prediction accuracy in the high concentration range of 6 –8 µg/ml (Fig. S4). As a result, the trained model predicts PM_c_ concentrations with a measurement error of 14.3 ± 15.4% (Fig. 5f, Table S7). However, without the correction process, the model fails to predict PM_c_ concentrations of PM mixture, as shown in Fig. 5g with gray rhombus symbols (Table S8).

Incorrect predictions of PM_c_ concentrations might occur because the low-pass filtered images of PM_c_ and PM_f_ look similar (Fig. 2). When low-pass filtered images of monodisperse PM_f_ are fed to the model trained with monodisperse PM_c_, the trained model incorrectly predicts PM_f_ concentrations as PM_c_ concentrations (*Y*) (Fig. 5d). The linear regression line of the mispredicted PM_c_ concentrations can be expressed as *Y* = *AX* + *B*, where *X* indicates the PM_f_ concentration. This result implies that LPF cannot fully filter PM_f_ signals from speckle images of PM mixture. Therefore, the PM_c_ concentrations predicted from low-pass filtered images of PM mixture (*P*) need to be corrected by subtracting the mispredicted PM_c_ concentration *Y*. In this study, two speckle signals of PM_f_ and PM_c_ acquired from PM mixture are assumed to overlap for providing a superposition signal. When PM_f_ and PM_c_ coexist in a 3D volume, multiple scattering phenomena occur successively by the presence of adjacent particles. However, an analytical estimation of the optical properties of the superposition signal is difficult to obtain due to the unpredictable volume scattering phenomena induced by polydisperse non-spherical PMs^43^. Therefore, the correction coefficient (*k*) should be multiplied to *Y* to consider the nonlinear superposition of two speckle signals of PM_f_ and PM_c_. The appropriate correction coefficient is estimated by searching the minimum RMSE of the corrected PM_c_ concentrations (*P*_*c*_ = *P* – *kY*) (Fig. 5e). Although the global minimum RMSE occurs at *R* = 15, the PM_f_ concentrations (*X*) should be accurately measured in advance. Therefore, the optimum *k* is set to 0.57 at *R* = 20. After the correction process, the performance of PM_c_ concentration prediction improves with a measurement error of 22.8 ± 18.1%, as illustrated in Fig. 5g with blue triangle symbols (Table S8).

## Discussion

Compared with conventional measurement techniques for particles, the proposed S-DHM device has a compact, cost-effective optical alignment composed of a 3D printed housing, an inexpensive laser diode, and commercial optical lenses. The proposed S-DHM device overcomes the concentration limit of commercially available hand-held particle counters used for PM monitoring (Table S9). The predictable range of PM size is limited by the spatial resolution (*Δ* = 0.61*λ*/NA = 811.3 nm) and the field-of-view (175 × 175 µm^2^) of the proposed S-DHM device. Since the magnified pixel length of the S-DHM device is 250 nm, it is possible for the image sensor of the smartphone to record PM signals up to the spatial resolution. The spatial resolution can be further enhanced with increasing the numerical aperture (NA) of the objective lens and decreasing the wavelength of the laser diode. By reducing the magnification of the objective lens and using an up-to-date smartphone whose image sensor has a larger number of pixels, the field-of-view of the S-DHM device can be further expanded. However, this kind of hardware upgrade will increase the total cost of the optical arrangement.

Recently, the specifications of hand-held smartphones have been remarkably improved. For example, the resolution of a smartphone image sensor exceeds 10 megapixels. The performance of a CPU embedded in a smartphone is sufficient to compute digital image processing and operate pretrained deep learning models using TensorFlow Lite. This implies that a smartphone can be utilized to replace the expensive camera of a conventional DHM system. However, the prediction accuracy of the S-DHM system still has technical limitations. Given that the exposure time of a smartphone camera is over 42 µs, the power of a laser diode cannot exceed 20 mW to avoid pixel saturation. The weak-powered laser beam generates holographic speckle patterns with a low SNR. The random motions of suspended PMs cause continuous fluctuations in the intensity of background noises. Therefore, sufficient removal of the background noises an ensemble averaging method becomes difficult, and the remaining background noises contained in all datasets with low SNRs can reduce the prediction accuracy. As a result, the predicted PM concentrations in the range of 1–2 µg/ml is found to be higher than the target value, whereas those of 7–8 µg/ml is lower (Figs. 4e, 5f). The accuracy of the proposed S-DHM device can be enhanced in the near future with further improvements in the hardware of smartphone camera and laser power.

Although the proposed device can measure PM_f_ concentrations of both monodisperse PM_f_ and PM mixture samples with a moderate accuracy, the measurement of PM_c_ concentration remains somewhat limited. Given the overlapping spatial frequency distributions of low-pass filtered PM_f_ and PM_c_ speckles, it is hard to fully separate the two speckles using a 2D Gaussian filter. To filter PM_f_ speckles with lower spatial frequency, the filter size should be increased. However, since some PM_c_ speckles are lost together, the measurement error rather increases (Fig. 5c,e). Therefore, an additional correction process is required. Due to the complex volume scattering phenomena of PMs, it is difficult to verify that the correction coefficient (*k* = 0.57) is theoretically acceptable. For example, the total RMSE of the measured PM_c_ concentrations is largely reduced by the adoption of correction process, while the measurement errors of several datasets with high PM_c_ concentration labels rather increases (Table S8). It implies that the correction coefficient (*k*) may have nonlinear relationships with the PM concentration ratios. For further improvement of the correction process, the correlation between the correction coefficient (*k*) and PM concentrations should be investigated in detail. To increase the prediction accuracy of PM_c_ concentration without correction process, there is an option to generate input datasets of all combinations of polydisperse PM mixtures and train deep learning algorithms. However, this approach might require cumbersome sample preparation and high computational cost compared with the proposed method.

## Conclusions

In summary, a hand-held smartphone-based device for cost-effective monitoring of suspended PMs in water is developed by integrating a S-DHM system and deep learning network. The developed device can selectively measure mass concentrations of PM_f_ and PM_c_ from a holographic speckle image of polydisperse PM mixture. The predictable particle size and concentration range of PMs can be extended by training the deep learning algorithms with additional datasets. The proposed PM monitoring technique can be applied to various micro-scale pollutants suspended in diverse media, such as microplastics and airborne PMs. This study would contribute to the development of compact hand-held devices for real-time monitoring of environmental quality with a portable smartphone.

## Supplementary Information

Supplementary Information.

## Data Availability

All data used to evaluate the results in the paper are present in the main text and/or the Supplementary Information. Additional data related to this paper may be available from the authors.

## References

[1] Engel-Cox J, Oanh NTK, van Donkelaar A, Martin RV, Zell E (2013). Toward the next generation of air quality monitoring: Particulate matter. Atmos. Environ..

[2] Altenburger R (2015). Future water quality monitoring—Adapting tools to deal with mixtures of pollutants in water resource management. Sci. Total Environ..

[3] Matos T (2019). Development of a cost-effective optical sensor for continuous monitoring of turbidity and suspended particulate matter in marine environment. Sensors.

[4] Zhang R (2018). Morphology and property investigation of primary particulate matter particles from different sources. Nano Res..

[5] Kelly FJ, Fussell JC (2012). Size, source and chemical composition as determinants of toxicity attributable to ambient particulate matter. Atmos. Environ..

[6] Di Q (2017). Air pollution and mortality in the medicare population. N. Engl. J. Med..

[7] Xi J (2018). Visualization of local deposition of nebulized aerosols in a human upper respiratory tract model. J. Vis..

[8] Bilotta GS (2012). Developing environment-specific water quality guidelines for suspended particulate matter. Water Res..

[9] Manjunatha B, Deekshitha B, Seo E, Kim J, Lee SJ (2021). Developmental toxicity induced by particulate matter (PM_2.5_) in zebrafish (*Danio rerio*) model. Aquat. Toxicol..

[10] Jiang ZP (2021). The effects of suspended particulate matter, nutrient, and salinity on the growth of *Amphidinium carterae* under estuary environmental conditions. Front. Mar. Sci..

[11] Barth HG (1984). Modern Methods of Particle Size Analysis.

[12] Shekunov BY, Chattopadhyay P, Tong HH, Chow AH (2007). Particle size analysis in pharmaceutics: Principles, methods and applications. Pharm. Ress.

[13] Kulkarni P, Baron PA, Willeke K (2011). Aerosol Measurement: Principles, Techniques, and Applications.

[14] Huang NT, Zhang HL, Chung M-T, Seo JH, Kurabayashi K (2014). Recent advancements in optofluidics-based single-cell analysis: Optical on-chip cellular manipulation, treatment, and property detection. Lab Chip.

[15] Shang J, Gao X (2014). Nanoparticle counting: Towards accurate determination of the molar concentration. Chem. Soc. Rev..

[16] Noble CA (2001). Federal reference and equivalent methods for measuring fine particulate matter. Aerosol Sci. Technol..

[17] Giddings JC, Yang FJ, Myers MN (1974). Sedimentation field-flow fractionation. Anal. Chem..

[18] Ma Z, Merkus HG, de Smet JG, Heffels C, Scarlett B (2000). New developments in particle characterization by laser diffraction: Size and shape. Powder Technol..

[19] Perkampus H-H (2013). UV-VIS Spectroscopy and its Applications.

[20] Filipe V, Hawe A, Jiskoot W (2010). Critical evaluation of nanoparticle tracking analysis (NTA) by NanoSight for the measurement of nanoparticles and protein aggregates. Pharm. Res..

[21] Dragovic RA (2011). Sizing and phenotyping of cellular vesicles using nanoparticle tracking analysis. Nanomed. Nanotechnol. Biol. Med..

[22] Stetefeld J, McKenna SA, Patel TR (2016). Dynamic light scattering: A practical guide and applications in biomedical sciences. Biophys. Rev..

[23] Austin J, Minelli C, Hamilton D, Wywijas M, Jones HJ (2020). Nanoparticle number concentration measurements by multi-angle dynamic light scattering. J. Nanoparticle Res..

[24] Langevin D (2018). Towards reproducible measurement of nanoparticle size using dynamic light scattering: Important controls and considerations. NanoImpact.

[25] Marucco A (2019). Applicability and limitations in the characterization of poly-dispersed engineered nanomaterials in cell media by dynamic light scattering (DLS). Materials.

[26] Malm AV, Corbett JC (2019). Improved dynamic light scattering using an adaptive and statistically driven time resolved treatment of correlation data. Sci. Rep..

[27] Park Y, Depeursinge C, Popescu G (2018). Quantitative phase imaging in biomedicine. Nat. Photonics.

[28] Zhu Y, Yeung CH, Lam EY (2021). Microplastic pollution monitoring with holographic classification and deep learning. J. Phys. Photonics.

[29] Wu YC (2017). Air quality monitoring using mobile microscopy and machine learning. Light Sci. Appl..

[30] Go T, Kim J, Lee SJ (2021). Three-dimensional volumetric monitoring of settling particulate matters on a leaf using digital in-line holographic microscopy. J. Hazard. Mater..

[31] Kim MK (2010). Principles and techniques of digital holographic microscopy. SPIE Rev..

[32] Memmolo P (2011). Automatic focusing in digital holography and its application to stretched holograms. Opt. Lett..

[33] Choi YS, Seo KW, Sohn MH, Lee SJ (2012). Advances in digital holographic micro-PTV for analyzing microscale flows. Opt. Lasers Eng..

[34] LeCun Y, Bengio Y, Hinton G (2015). Deep learning. Nature.

[35] Byeon H, Go T, Lee SJ (2019). Deep learning-based digital in-line holographic microscopy for high resolution with extended field of view. Opt. Laser Technol..

[36] Go T, Yoon GY, Lee SJ (2019). Learning-based automatic sensing and size classification of microparticles using smartphone holographic microscopy. Analyst.

[37] Shao S, Mallery K, Kumar SS, Hong J (2020). Machine learning holography for 3D particle field imaging. Opt. Express.

[38] Rivenson Y, Wu Y, Ozcan A (2019). Deep learning in holography and coherent imaging. Light Sci. Appl..

[39] Meng X (2017). Smartphone based hand-held quantitative phase microscope using the transport of intensity equation method. Lab Chip.

[40] Wu Y, Ozcan A (2018). Lensless digital holographic microscopy and its applications in biomedicine and environmental monitoring. Methods.

[41] Kim J, Go T, Lee SJ (2021). Volumetric monitoring of airborne particulate matter concentration using smartphone-based digital holographic microscopy and deep learning. J. Hazard. Mater..

[42] Piederrière Y (2005). Backscattered speckle size as a function of polarization: Influence of particle-size and-concentration. Opt. Express.

[43] Goodman JW (2007). Speckle Phenomena in Optics: Theory and Applications.

[44] Hinton G, Salakhutdinov R (2012). An efficient learning procedure for deep Boltzmann machines. Neural Comput..

[45] Pan B, Lu Z, Xie H (2010). Mean intensity gradient: An effective global parameter for quality assessment of the speckle patterns used in digital image correlation. Opt. Lasers Eng..

[46] Alexander TL, Harvey JE, Weeks AR (1994). Average speckle size as a function of intensity threshold level: Comparison of experimental measurements with theory. Appl. Opt..

[47] Piederrière Y (2004). Scattering through fluids: Speckle size measurement and Monte Carlo simulations close to and into the multiple scattering. Opt. Express.

