# Supplementary material for: Smartphone-based holographic measurement of polydisperse suspended particulate matter with various mass concentration ratios
Source: Sci Rep. 2022 Dec 30;12:22609. doi: 10.1038/s41598-022-27215-6 (PMC9803653; doi:10.1038/s41598-022-27215-6)
Supplement: Supplementary file 1 — Supplementary Information. [file 41598_2022_27215_MOESM1_ESM.pdf]

Supplementary Information

Smartphone-based holographic measurement of polydisperse suspended particulate matter with various mass concentration ratios

Jihwan Kim[1], Youngdo Kim[1], Kyler J. Howard[2], Sang Joon Lee[1*]

[1]Department of Mechanical Engineering, Pohang University of Science and Technology, Pohang, 37673, Republic of Korea

[2]School of Biomedical Engineering, Colorado State University, Fort Collins, Colorado, 80521, USA

*Corresponding Author: **Prof. Sang Joon Lee**

Department of Mechanical Engineering, Pohang University of Science and Technology, Pohang, 37673, Republic of Korea

E-mail: sjlee@postech.ac.kr

Phone: +82-54-279-2169

Fax: +82-54-279-3199

Table S1. Mean intensity graidents of holographic speckle patterns acquired from PM samples with various concentration ratios (Fig. 2b).

PM concentration (μg/ml)			Mean intensity gradient (Δ*I*/px)	
	ρ_f	ρ_c	HPF	LPF
PM_f samples	1	0	0.01162 ± 0.00043	0.00293 ± 0.00007
	2	0	0.01201 ± 0.00076	0.00350 ± 0.00006
	3	0	0.01574 ± 0.00070	0.00418 ± 0.00004
	4	0	0.01528 ± 0.00106	0.00455 ± 0.00009
	5	0	0.01834 ± 0.00086	0.00507 ± 0.00009
	6	0	0.01785 ± 0.00096	0.00593 ± 0.00010
	7	0	0.01957 ± 0.00078	0.00610 ± 0.00008
	8	0	0.02214 ± 0.00134	0.00695 ± 0.00011
PM_c samples	0	1	0.01003 ± 0.00016	0.00249 ± 0.00005
	0	2	0.00929 ± 0.00042	0.00304 ± 0.00022
	0	3	0.00691 ± 0.00034	0.00242 ± 0.00009
	0	4	0.00980 ± 0.00036	0.00305 ± 0.00010
	0	5	0.00994 ± 0.00068	0.00344 ± 0.00010
	0	6	0.00898 ± 0.00048	0.00346 ± 0.00012
	0	7	0.00812 ± 0.00056	0.00367 ± 0.00025
	0	8	0.00953 ± 0.00108	0.00447 ± 0.00025
PM mixture samples	1.5	1.5	0.01216 ± 0.00053	0.00354 ± 0.00005
	1.75	3.25	0.01028 ± 0.00078	0.00379 ± 0.00009
	2	4	0.01185 ± 0.00088	0.00471 ± 0.00015
	2.5	2.5	0.01319 ± 0.00084	0.00426 ± 0.00011
	2.75	4.25	0.01380 ± 0.00099	0.00476 ± 0.00008
	3	3	0.01364 ± 0.00086	0.00470 ± 0.00009
	3.25	1.75	0.01361 ± 0.00190	0.00446 ± 0.00040
	3.5	3.5	0.01362 ± 0.00098	0.00492 ± 0.00009
	4	2	0.01510 ± 0.00170	0.00522 ± 0.00025
	4.25	2.75	0.01581 ± 0.00108	0.00518 ± 0.00011
	4.5	4.5	0.01843 ± 0.00116	0.00557 ± 0.00007
	3	7	0.01187 ± 0.00210	0.00560 ± 0.00035
	5	5	0.01355 ± 0.00182	0.00631 ± 0.00033
	7	3	0.01810 ± 0.00169	0.00686 ± 0.00013
	6	6	0.01657 ± 0.00126	0.00648 ± 0.00013
	7	7	0.02147 ± 0.00212	0.00676 ± 0.00029

Table S2. Speckle sizes of holographic speckle patterns acquired from PM samples with various concentration ratios (Fig. 2c).

PM concentration (μg/ml)			Speckle size (px)	
	ρ_f	ρ_c	HPF	LPF
PM_f samples	1	0	5.270 ± 0.248	66.713 ± 6.604
	2	0	7.237 ± 0.241	94.749 ± 5.665
	3	0	9.094 ± 0.275	180.433 ± 30.462
	4	0	11.369 ± 0.406	135.984 ± 11.195
	5	0	12.830 ± 0.584	184.512 ± 10.660
	6	0	14.703 ± 0.561	232.416 ± 19.031
	7	0	15.387 ± 0.703	237.547 ± 13.150
	8	0	19.568 ± 0.808	257.244 ± 14.988
PM_c samples	0	1	5.502 ± 0.246	84.596 ± 9.910
	0	2	11.676 ± 1.984	155.484 ± 30.295
	0	3	5.464 ± 0.378	331.826 ± 137.724
	0	4	9.132 ± 0.933	295.151 ± 46.774
	0	5	10.950 ± 0.665	499.733 ± 91.090
	0	6	11.069 ± 1.092	452.859 ± 64.926
	0	7	10.862 ± 1.351	789.239 ± 197.001
	0	8	14.868 ± 2.300	827.967 ± 138.001
PM mixture samples	1.5	1.5	6.324 ± 0.178	224.519 ± 24.000
	1.75	3.25	7.182 ± 0.234	242.453 ± 35.094
	2	4	8.914 ± 0.476	356.397 ± 49.765
	2.5	2.5	8.832 ± 0.353	242.546 ± 55.327
	2.75	4.25	10.064 ± 0.308	322.187 ± 36.380
	3	3	9.769 ± 0.328	258.330 ± 30.651
	3.25	1.75	9.631 ± 1.675	209.852 ± 32.006
	3.5	3.5	10.576 ± 0.459	295.496 ± 33.074
	4	2	12.243 ± 0.869	215.438 ± 28.147
	4.25	2.75	12.249 ± 0.499	283.264 ± 41.801
	4.5	4.5	13.340 ± 0.613	324.425 ± 29.590
	3	7	10.170 ± 1.325	548.199 ± 75.563
	5	5	11.837 ± 1.003	529.508 ± 68.193
	7	3	16.591 ± 0.563	355.615 ± 32.031
	6	6	14.184 ± 0.392	457.625 ± 52.258
	7	7	17.254 ± 1.450	429.651 ± 53.955

Table S3. Speckle widths of holographic speckle patterns acquired from PM samples with various concentration ratios (Fig. 2d).

PM concentration (μg/ml)			Speckle width (μm)	
	ρ_f	ρ_c	HPF	LPF
PM_f samples	1	0	0.859 ± 0.014	3.935 ± 0.123
	2	0	0.905 ± 0.015	4.042 ± 0.094
	3	0	0.887 ± 0.013	5.001 ± 0.808
	4	0	0.941 ± 0.036	3.880 ± 0.111
	5	0	0.907 ± 0.029	4.095 ± 0.124
	6	0	0.959 ± 0.039	4.036 ± 0.222
	7	0	0.940 ± 0.027	3.978 ± 0.154
	8	0	0.953 ± 0.037	3.791 ± 0.102
PM_c samples	0	1	0.923 ± 0.023	5.000 ± 0.295
	0	2	0.923 ± 0.031	5.704 ± 0.672
	0	3	1.070 ± 0.217	8.434 ± 2.026
	0	4	0.943 ± 0.022	7.278 ± 0.480
	0	5	0.955 ± 0.056	8.227 ± 0.535
	0	6	0.923 ± 0.044	8.382 ± 1.504
	0	7	0.976 ± 0.052	9.392 ± 2.827
	0	8	1.005 ± 0.054	8.831 ± 1.981
PM mixture samples	1.5	1.5	0.884 ± 0.052	6.132 ± 0.264
	1.75	3.25	0.980 ± 0.101	5.759 ± 0.349
	2	4	0.993 ± 0.055	6.139 ± 0.532
	2.5	2.5	0.923 ± 0.023	5.560 ± 0.567
	2.75	4.25	0.948 ± 0.045	5.760 ± 0.343
	3	3	0.950 ± 0.025	5.344 ± 0.285
	3.25	1.75	0.940 ± 0.045	5.156 ± 0.518
	3.5	3.5	0.972 ± 0.031	5.537 ± 0.364
	4	2	0.977 ± 0.051	4.629 ± 0.276
	4.25	2.75	0.952 ± 0.047	5.297 ± 0.273
	4.5	4.5	0.919 ± 0.040	5.299 ± 0.257
	3	7	1.065 ± 0.164	6.300 ± 0.669
	5	5	1.049 ± 0.109	5.728 ± 0.755
	7	3	1.013 ± 0.095	4.696 ± 0.176
	6	6	1.007 ± 0.101	5.388 ± 0.267
	7	7	0.937 ± 0.068	5.247 ± 0.331

Table S4. Spatial frequencies of holographic speckle patterns acquired from PM samples with various concentration ratios (Fig. 2e).

PM concentration (μg/ml)			Spatical frequency (cycles/px)	
	ρ_f	ρ_c	HPF	LPF
PM_f samples	1	0	0.09304 ± 0.00435	0.01882 ± 0.00162
	2	0	0.10202 ± 0.00384	0.01836 ± 0.00176
	3	0	0.10278 ± 0.00425	0.01162 ± 0.00464
	4	0	0.08452 ± 0.00632	0.01826 ± 0.00173
	5	0	0.09883 ± 0.00364	0.01495 ± 0.00225
	6	0	0.09292 ± 0.00349	0.01679 ± 0.00310
	7	0	0.09770 ± 0.00321	0.01891 ± 0.00169
	8	0	0.09578 ± 0.00397	0.01952 ± 0.00157
PM_c samples	0	1	0.08534 ± 0.01125	0.01363 ± 0.00149
	0	2	0.08372 ± 0.00570	0.01174 ± 0.00275
	0	3	0.08428 ± 0.01061	0.00799 ± 0.00199
	0	4	0.08650 ± 0.00473	0.00873 ± 0.00145
	0	5	0.08275 ± 0.00539	0.00760 ± 0.00115
	0	6	0.08854 ± 0.00514	0.00748 ± 0.00146
	0	7	0.08538 ± 0.00433	0.00591 ± 0.00213
	0	8	0.08591 ± 0.00539	0.00692 ± 0.00169
PM mixture samples	1.5	1.5	0.09823 ± 0.00586	0.01020 ± 0.00116
	1.75	3.25	0.07911 ± 0.01203	0.01168 ± 0.00151
	2	4	0.08816 ± 0.00801	0.00983 ± 0.00176
	2.5	2.5	0.08534 ± 0.01125	0.01232 ± 0.00244
	2.75	4.25	0.07844 ± 0.00832	0.01154 ± 0.00170
	3	3	0.09732 ± 0.00384	0.01239 ± 0.00173
	3.25	1.75	0.08593 ± 0.00563	0.01204 ± 0.00218
	3.5	3.5	0.07840 ± 0.00832	0.01152 ± 0.00164
	4	2	0.08763 ± 0.00575	0.01395 ± 0.00192
	4.25	2.75	0.08992 ± 0.00505	0.01018 ± 0.00214
	4.5	4.5	0.09381 ± 0.00464	0.01190 ± 0.00151
	3	7	0.08305 ± 0.01078	0.01036 ± 0.00186
	5	5	0.08435 ± 0.00865	0.01107 ± 0.00205
	7	3	0.08206 ± 0.00966	0.01425 ± 0.00163
	6	6	0.08623 ± 0.00556	0.01193 ± 0.00170
	7	7	0.09268 ± 0.01083	0.01184 ± 0.00170

Table S5. Predicted PM_f concentrations and measurement accuracies of monodisperse PM_f samples (Fig. 4e).

	Ground truth PM_f concentration (μg/ml)	Predicted PM_f concentration (μg/ml)	Measurement error (%)
PM_f samples	1	1.529 ± 0.127	52.9
	2	2.073 ± 0.197	3.6
	3	3.540 ± 0.211	18.0
	4	4.002 ± 0.364	0.1
	5	5.341 ± 0.197	6.8
	6	5.643 ± 0.227	5.9
	7	6.006 ± 0.423	14.2
	8	7.115 ± 0.258	11.1

Table S6. Predicted PM_f concentrations and measurement accuracies of polydisperse PM mixture samples (Fig. 4f).

	PM concentration (μg/ml)		Predicted PM_f concentration (μg/ml)		Measurement error (%)	
	ρ_f	ρ_c	w/o HPF	w/ HPF	w/o HPF	w/ HPF
PM mixture samples	1.5	1.5	3.531 ± 0.136	1.818 ± 0.075	135.4	21.2
	1.75	3.25	3.996 ± 0.201	1.839 ± 0.112	128.4	5.1
	2	4	5.441 ± 0.232	2.653 ± 0.450	172.0	32.6
	2.5	2.5	4.640 ± 0.291	2.877 ± 0.456	85.6	15.1
	2.75	4.25	5.439 ± 0.141	3.584 ± 0.235	97.8	30.3
	3	3	5.331 ± 0.190	3.585 ± 0.190	77.7	19.5
	3.25	1.75	4.765 ± 0.117	3.166 ± 0.318	46.6	2.6
	3.5	3.5	5.561 ± 0.141	3.671 ± 0.244	58.9	4.9
	4	2	5.738 ± 0.117	4.487 ± 0.324	43.5	12.2
	4.25	2.75	5.777 ± 0.173	4.443 ± 0.578	35.9	4.5
	4.5	4.5	6.229 ± 0.113	5.421 ± 0.214	38.4	20.5
	3	7	6.485 ± 0.102	2.974 ± 0.289	116.2	0.9
	5	5	6.949 ± 0.082	3.977 ± 0.382	39.0	20.5
	7	3	7.079 ± 0.100	6.157 ± 0.442	1.1	12.0
	6	6	6.976 ± 0.103	5.444 ± 0.361	16.3	9.3
	7	7	7.001 ± 0.058	6.610 ± 0.262	0.0	5.6

Table S7. Predicted PM_c concentrations and measurement accuracies of monodisperse PM_c samples (Fig. 5f).

	Ground truth PM_c concentration (μg/ml)	Predicted PM_c concentration (μg/ml)	Measurement error (%)
PM_c samples	1	1.416 ± 0.072	41.6
	2	2.426 ± 0.267	21.3
	3	2.742 ± 0.197	8.6
	4	3.768 ± 0.357	5.8
	5	5.084 ± 0.316	1.7
	6	5.138 ± 0.276	14.4
	7	5.886 ± 0.517	15.9
	8	6.854 ± 0.191	14.3

Table S8. Predicted PM_c concentrations and measurement accuracies of polydisperse PM mixture samples (Fig. 5g).

	PM concentration (μg/ml)		Predicted PM_c concentration (μg/ml)		Measurement error (%)	
	ρ_f	ρ_c	w/o correction	w/ correction	w/o correction	w/ correction
PM mixture samples	1.5	1.5	3.582 ± 0.242	2.488 ± 0.242	138.8	65.9
	1.75	3.25	4.341 ± 0.344	3.171 ± 0.344	33.6	2.4
	2	4	5.809 ± 0.329	4.133 ± 0.329	45.2	3.3
	2.5	2.5	4.586 ± 0.543	2.999 ± 0.543	83.4	20.0
	2.75	4.25	5.663 ± 0.239	3.911 ± 0.239	33.3	8.0
	3	3	5.269 ± 0.332	3.489 ± 0.332	75.6	16.3
	3.25	1.75	4.312 ± 0.433	2.600 ± 0.433	146.4	48.6
	3.5	3.5	5.541 ± 0.265	3.739 ± 0.265	58.3	6.8
	4	2	4.957 ± 0.486	2.771 ± 0.486	147.8	38.5
	4.25	2.75	5.439 ± 0.317	3.233 ± 0.317	97.8	17.6
	4.5	4.5	6.210 ± 0.221	3.834 ± 0.221	38.0	14.8
	3	7	7.100 ± 0.265	5.359 ± 0.265	1.4	23.4
	5	5	7.295 ± 0.257	5.249 ± 0.257	45.9	5.0
	7	3	6.871 ± 0.179	4.113 ± 0.179	129.0	37.1
	6	6	7.229 ± 0.163	4.847 ± 0.163	20.5	19.2
	7	7	7.143 ± 0.236	4.391 ± 0.236	2.0	37.3

Table S9. Comparison of concentration limits and the corresponding coincidence errors of commercial hand-held particle counters used for PM monitoring. Assuming that the PM density is 2,700 kg/m^3 and PM particles are spherical, the concentration limit in the unit of particles per cubic meter is converted into the mass concentration in the unit of micrograms per mililiter. The diameters of PM_c and PM_f particles are set to 10 and 2.5 μm, respectively. Since the PM concentrations tested in this study are 1 ~ 8 μg/ml, the proposed device can measure much higher concentration range with a moderate measurement accuracy, compared to the commercially available hand-held particle counters.

		**Product A[1]**	**Product B[2]**	**Product C[3]**
**Concentration limit (particles/m^3)**		210,000,000	141,257,902	70,000,000
**Concentration limit (μg/ml)**	PM_c	0.2967	0.1996	0.0989
	PM_f	0.0046	0.0031	0.0015
**Coincidence error (%)**		10	10	5

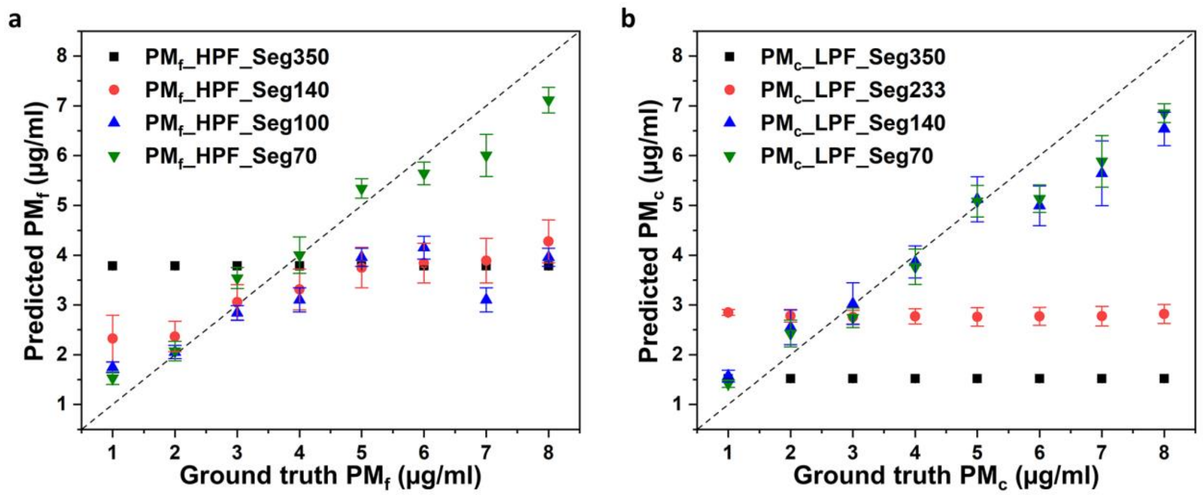

Figure S1. Comparison of the predicted mass concentrations according to the size of segmented images. (a) High-pass filtered holograms of monodisperse PM_f are segmented into 350×350, 140×140, 100×100, and 70×70 pixels. (b) Low-pass filtered holograms of monodisperse PM_c are segmented into 350×350, 233×233, 140×140, and 70×70 pixels.

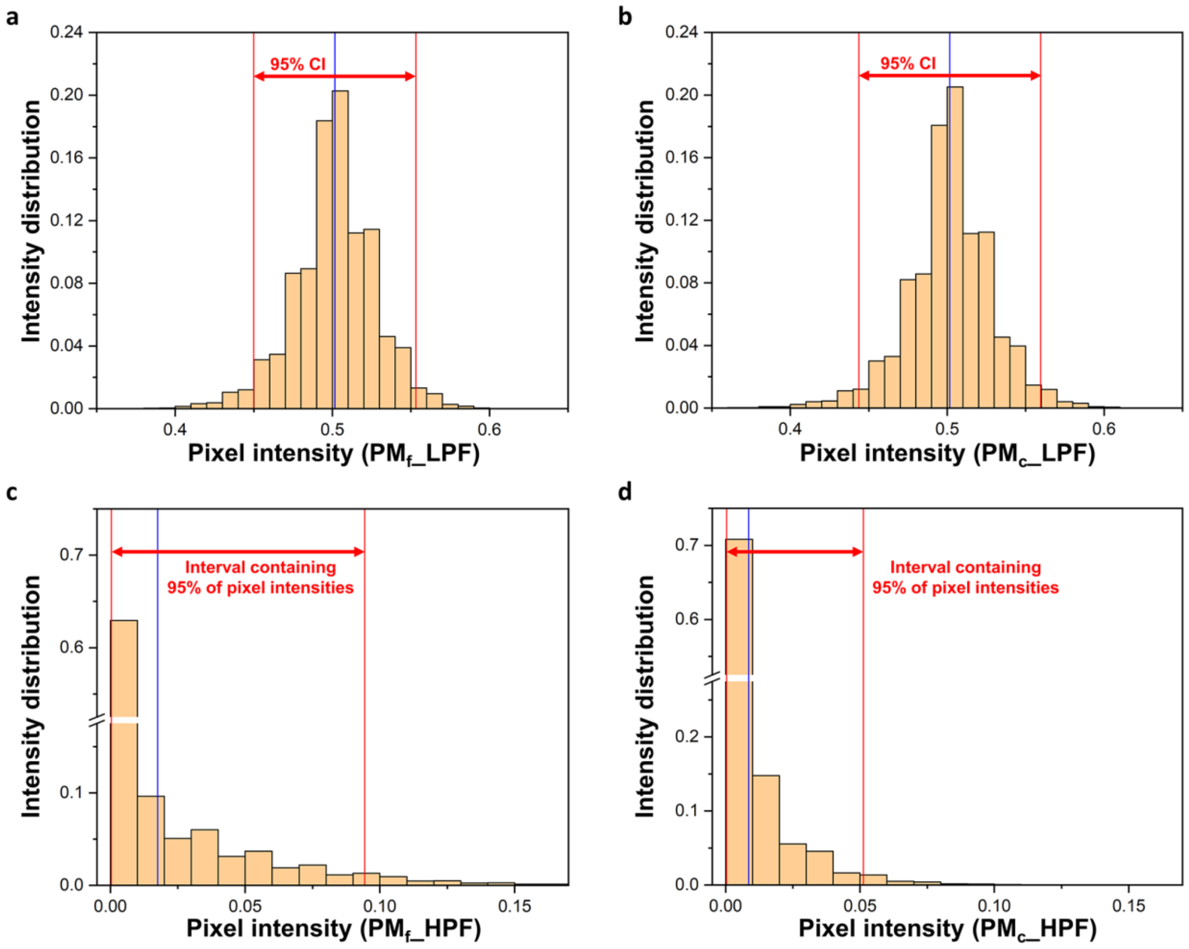

Figure S2. Normalized intensity distributions of holographic speckle patterns: (a, b) low-pass filtered holograms of monodisperse PM_f and PM_c, and (c, d) high-pass filtered holograms of monodisperse PM_f and PM_c. The blue line of each case indicates the average pixel intensity. The red lines in (a, b) and (c, d) denote the 95% confidence intervals (CI) and the intervals containing 95% of pixel intensities, respectively.

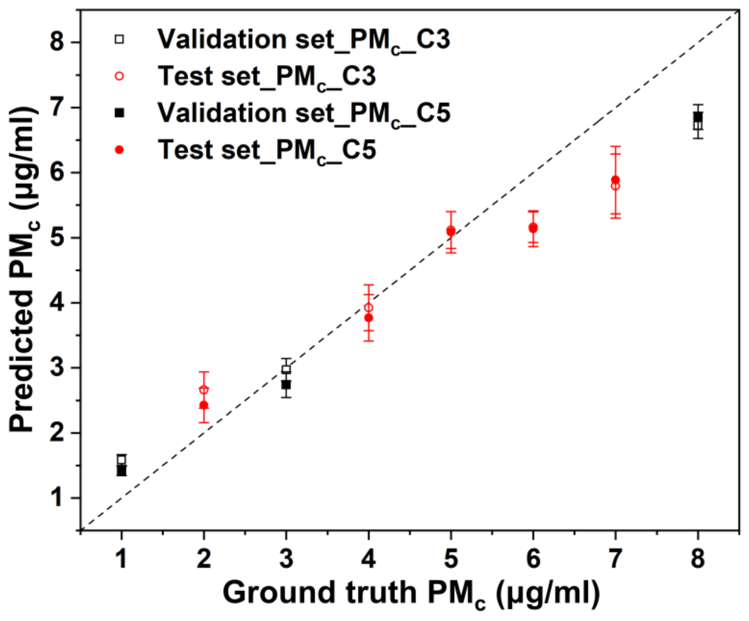

Figure S3. Comparison of the predicted mass concentrations of monodisperse PM_c samples with the corresponding ground truth concentrations for the contrast enhancement parameter *C* of 3 and 5.

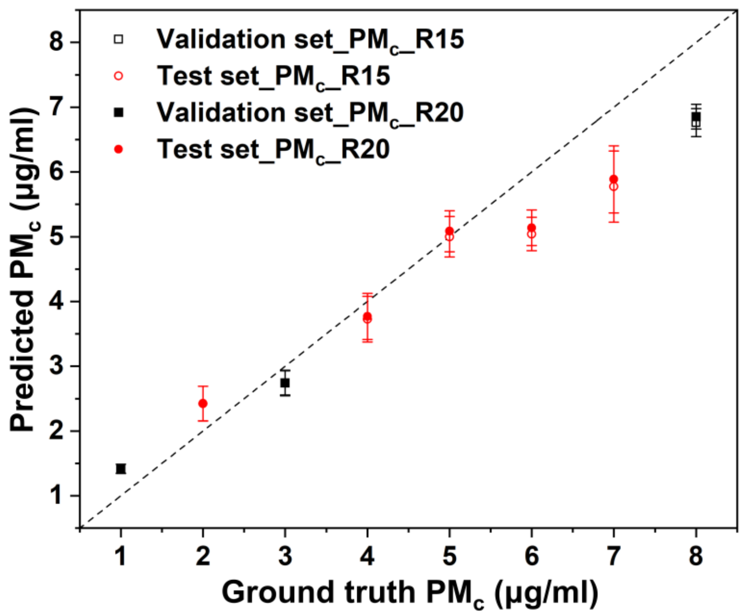

Figure S4. Comparison of the predicted mass concentrations of monodisperse PM_c samples with the corresponding ground truth concentrations for the Gaussian filter size *R* of 15 and 20.

References

1. AeroTrak handheld particle counter 9306, TSI, USA, Available at: https://tsi.com/aerotrak-handheld-particle-counter-9306/ (Accessed 22 October 2022).

2. MET ONE HHPC 6+, Beckman Coulter, USA, Available at: https://www.beckman.kr/air-particle-counters/met-one-hhpc-plus-series (Accessed 22 October 2022).

3. TES-5200 particle mass counter, TES Electrical Electronic Corporation, Available at: http://www.tes.com.tw/en/product_detail.asp?seq=423 (Accessed 22 October 2022).
